# Deep Eutectic Polymer Electrolyte with Competitive Hydrogen‐Bonding Coordination for High‐Voltage Nickel‐rich Lithium Metal Batteries

**DOI:** 10.1002/advs.75883

**Published:** 2026-05-30

**Authors:** Yuxin Fan, Miao He, Yin Hu, Wei Chen, Yichao Yan, Tianyu Lei, Dongjiang Chen

**Affiliations:** ^1^ State Key Laboratory of Electronic Thin Films and Integrated Devices University of Electronic Science and Technology of China Chengdu China

**Keywords:** DES polymer electrolytes, high rate, Li metal battery, NCM811 cathodes stability

## Abstract

The pursuit of high‐energy‐density solid‐state batteries using Li metal anodes and high‐voltage Ni‐rich cathodes is hindered by severe interfacial degradation. Conventional polymer electrolytes with electronegative groups strongly adsorb high‐valent nickel, accelerating cathode decomposition and oxygen release. Here, we develop a deep‐eutectic polymer electrolyte (p‐DEPE) via in situ copolymerization of cyanoacrylate and butyl acrylate within a LiTFSI/LiDFOB dual‐salt network to reshape the interfacial chemistry. This design creates an intermolecular hydrogen‐bonding matrix that establishes a competitive coordination environment at the cathode interface, effectively weakening Ni^4+^ adsorption on electronegative sites. The suppression of high‐value Ni inhibits the growth of a high‐resistance cathode–electrolyte interphase and retards the detrimental phase transition from a layered to a rock‐salt structure. Furthermore, the locally confined interaction between the cyano‐group and the cathode surface at high states of charge minimizes parasitic chemical reactions with lattice oxygen, thereby substantially reducing oxygen release. Consequently, Li||LiNi_0.8_Co_0.1_Mn_0.1_O_2_ cells with p‐DEPE deliver outstanding high‐rate performance, cycling over 200 cycles at 2 C at room temperature and at 3 C at 70°C. Moreover, a 4.5 V high‐loading Li||NCM811 pouch cell retains 97.3% of its initial capacity after 100 cycles. This work demonstrates a scalable polymer electrolyte strategy for high‐energy‐density lithium metal batteries.

## Graphical abstract

To stabilize high‐voltage Li||NCM811 batteries, we develop an in situ polymerized deep‐eutectic electrolyte (p‐DEPE) featuring competitive hydrogen‐bonding coordination. This design weakens Ni^4^^+^ adsorption, suppresses oxygen release, and enhances interfacial stability. The p‐DEPE enables outstanding rate capability and long cycle life, including 200 cycles at 2 C (25°C) and 300 cycles at 3 C (70°C).

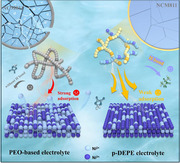

## 1 Introduction

The global transition toward sustainable energy has spurred an urgent demand for high‐energy‐density storage systems. Lithium metal batteries employing nickel‐rich layered oxide cathodes (LiNi_0_._8_Co_0_._1_Mn_0_._1_O_2_, NCM811) represent a promising pathway to meet this need. However, their practical deployment, especially in extreme‐environment applications such as aerospace and defense, is hampered by severe interfacial instabilities under high‐voltage operation [1, 2, 3]. Layered NCM811 owns a higher discharge capacity and the volume change is less than 2% during Li insertion/extraction [4, 5, 6, 7]. Charging NCM811 beyond 4.3 V versus Li^+^/Li, while necessary to unlock its full capacity, triggers detrimental surface reconstructions, including cationic mixing and phase transitions from a layered to spinel or rock‐salt structure [8, 9]. These processes are accompanied by the release of oxygen [10], which reacts aggressively with organic electrolytes, leading to gas evolution, rapid capacity fade, and significant safety risks [11, 12, 13]. Furthermore, the high reactivity of Ni^4^^+^ promotes the formation of a resistive NiO‐like layer at the cathode surface, which hinders the Li^+^ transportation and accelerates the capacity degradation [14]. Therefore, enabling Ni‐rich cathode materials with both high discharge capacity and stability necessitates suppressing the oxygen release and establishing the stable cathode–electrolyte interface (CEI) with less consumption of Ni^4+^ [15].

Polymer electrolytes have proved to be effective in high‐energy‐density batteries due to the excellent thermostability and low electrode interface resistance. However, traditional polymer electrolytes are featured with unstable high‐voltage cycling performance owing to the irreversible side reactions at the electrolyte/cathode interface [16], especially the PEO‐based polymer electrolytes. The inherent low oxidative potential of ether oxygen (EO) chains accelerates the polymer decomposition, triggering direct contact with the anode and cell failure [17, 18, 19].

Therefore, researchers have turned their attention to certain polymers containing specific functional groups. Because of carrying the highly polar functional group of ethyl cyanoacrylate (ECA), it not only promotes the dissociation of lithium salts but also enhances the oxidative stability of the polymer. Meanwhile, butyl acrylate (BA) has a lower glass transition temperature, which plays a positive role in improving the ionic conductivity of the polymer. Based on these outstanding characteristics, previous work [20] used ethyl cyanoacrylate (ECA) and butyl acrylate (BA) to form a dual continuous phase separated structure comprising a cyanide‐containing copolymer and a plastic crystal. While this design enhanced ionic conductivity (9.8 × 10^−4^ S cm^−1^ at 30°C) and high voltage stability (high cut‐off voltage of 4.7 V versus Li/ Li^+^) via the cyanide phase, it did not investigate how intermolecular interactions between the polymer and Li salts influence cathode stability or suppress nickel migration.

Besides, the strategies that focus on modulating the coordination environment within the electrolyte to improve the electrochemical stability at high voltage have been adopted [21, 22]. In addition, deep eutectic electrolytes (DEEs), composed of hydrogen bond donors and hydrogen bond acceptors, exhibit favorable interfacial wettability and weaken their solvating ability toward high‐valent transition metals. The electron‐deficient state in the nickel atom center is beneficial for improving the material stability owing to the coordination between electron‐absorbing groups and nickel atoms [23, 24], thereby passivating the cathode surface [25, 26, 27] [21, 28, 29]. However, DEEs suffer from insufficient Li^+^ conductivity and poor compatibility with lithium metal [30, 31]. Nevertheless, integrating high ionic conductivity with simultaneous stability toward both the lithium anode and high‐voltage cathode remains a formidable challenge [10, 32]. Combining solid polymer electrolytes with DEE concepts offers a promising route to overcome these limitations [33], yielding deep‐eutectic polymer electrolytes (DEPEs) with enhanced ionic transport and interfacial compatibility. Liu et al. [34] encapsulated 3,3′‐[oxydibis(2,1‐ethoxy)] dipropionitrile in polybutyl acrylate to fabricate an in situ DEPE. Their work focused on tuning solvation structures to generate dense SEI/CEI layers, but did not explore the direct impact of the DEPE on cathode stability, nor the underlying mechanism for suppressing nickel‐induced degradation.

In typical PDEEs, hydrogen bond donors are not incorporated as polymerizable monomers [35]. They simply act as plasticizers or crosslinkers. Huang [35] used the N‐(2‐Hydroxyethyl) acrylamide (HEAA) as a hydrogen bond donor to participate in the polymerization of DES. The cross‐linked polymer DES exhibits excellent high‐ion conductivity due to the intermolecular hydrogen bonding between the hydrogen bond donors and the Li salts, but its compatibility of NCM cathodes still remains to be explored. Although such systems achieve high ionic conductivity, their compatibility with Ni‐rich NCM cathodes and their ability to mitigate cathode interfacial degradation remain unexplored.

**FIGURE 1:**
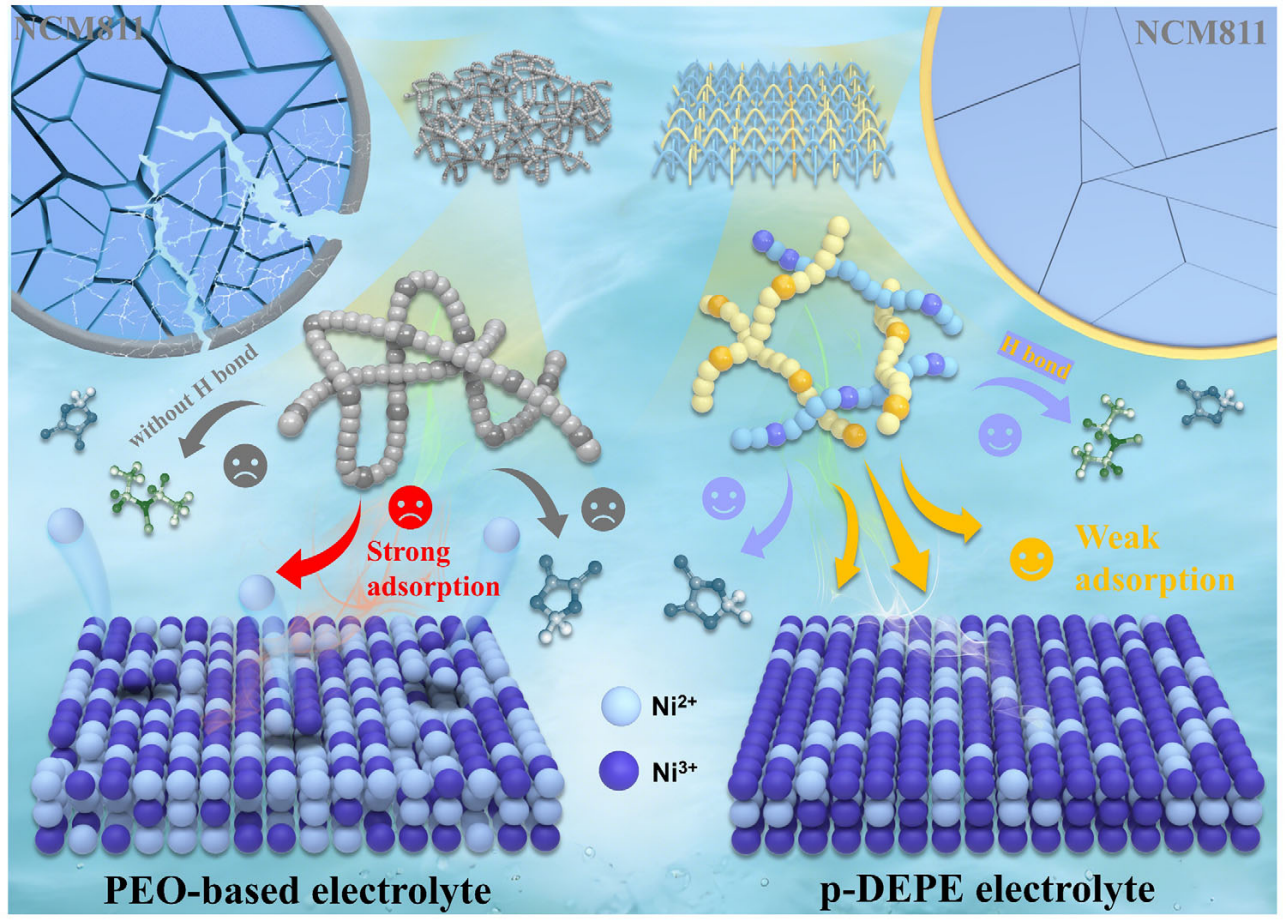
Conceptual design of polymerized deep‐eutectic polymer electrolyte (p‐DEPE) for suppressing the strong adsorption of high‐valent Ni species through competitive hydrogen‐bonding coordination.

Herein, we report an in situ polymerized deep‐eutectic polymer electrolyte (p‐DEPE) for stable high‐voltage Li||NCM811 batteries. P‐DEPE was synthesized from cyanoacrylate and butyl acrylate monomers in a dual‐salt (LiTFSI/LiDFOB) matrix. The intermolecular hydrogen‐bonding network formed between polymerizable ECA (as a hydrogen‐bond donor) and LiTFSI creates a competitive coordination environment that directly weakens the adsorption of high‐valent Ni^4+^ on electronegative groups. This suppression of nickel migration into the polymer is crucial for reducing Li/Ni mixing, mitigating lattice distortion, and preventing oxygen release, none of which were addressed in previous ECA‐BA or DEPE works. In addition, in our pDEPE, ECA acts both as a polymerizable monomer and a hydrogen bond donor, ensuring that the hydrogen bonding network is covalently integrated into the polymer backbone. This design maximizes atomic utilization and creates a stable, built in coordination environment, in contrast to prior PDEEs where donors are non‐polymerizable and prone to leaching (Figure 1). This unique deep eutectic structure facilitates rapid Li^+^ transport, achieving high ionic conductivity (0.6 mS cm^−1^ at 25°C) and anodic stability up to 5.2 V. With the p‐DEPE, a 4.5 V Li||NCM811 batteries achieve prolonged operation with a retention rate of 80% after 200 cycles at 2 C and exhibits outstanding thermostability over 300 cycles with the 80% capacity retention under 70°C at 3 C. Moreover, high‐loading roll cells (1.8 mAh cm^−2^) with the current density of exhibit good cycling stability. This work provides new insights into polymer design that concurrently addresses interfacial instability and ionic transport limitations, paving the way toward practical high‐voltage and high‐rate Li metal batteries.

## 2 Results and Discussion

### 2.1 Identifying PEO‐Induced Nickel Consumption at High Voltage

The development of high‐voltage stable electrolytes compatible with Ni‐rich cathodes requires a fundamental understanding of their interfacial failure mechanisms, particularly under charging conditions beyond 4.2 V. Nickel (Ni) dissolution from the surface of NCM811 particles significantly accelerates the interface side reactions under higher voltage (> 4.5 V), leading to the electrolyte decomposition, oxygen loss, and capacity fading [36, 37]. In addition, the awful side reactions induce severe interface degradation after long cycling [17, 38].

**FIGURE 2:**
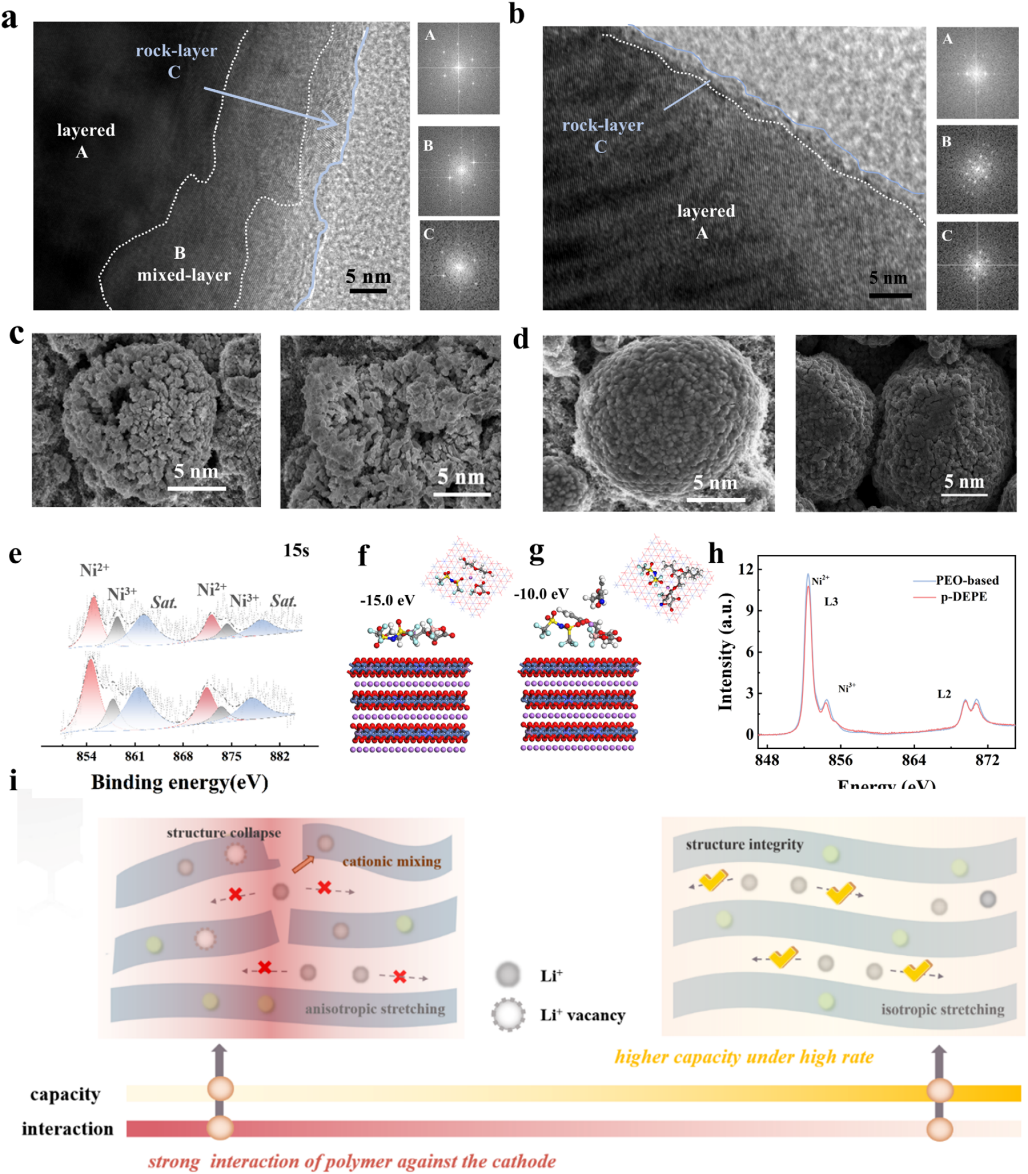
The TEM profiles of (a) PEO‐based and (b) p‐DEPE samples. The SEM profiles of (c) PEO‐based and (d) p‐DEPE samples. (e) The XPS profiles of p‐DEPE and PEO‐based samples after 15 s Ar sputtering. The adsorption energy of (f) PEO and (g) p‐DEPE on the surface of NCM811 cathodes. (h) The XAS images of PEO‐based and p‐DEPE samples. (i) Schematic diagram of p‐DEPE and PEO‐based samples in weakening the solvating ability of electronegative groups for high valence Ni at high voltage.

To elucidate the mechanism of interface failure and cathode decomposition, we first employed transmission electron microscopy (TEM) to examine the phase evolution of NCM811 particles after high‐voltage cycling. As shown in Figure 2a, cathodes cycled with PEO‐based electrolyte exhibit a pronounced phase transition from a layered to a rock‐salt structure, with a notably thick disordered layer at the surface [17]. This transformation stems from the chemical instability of Ni^4^^+^ under highly delithiated conditions, coupled with the strong adsorption of electronegative polymer functional groups onto nickel species. This interaction promotes Ni migration from the cathode surface into the electrolyte, where it is reduced to lower valence states, triggering interfacial decomposition and structural collapse [25, 39]. Consequently, designing a high‐voltage‐stable electrolyte requires suppressing the migration of high‐valent nickel and weakening the unfavorable polymer–cathode interaction.

In contrast, the polyacrylate‐based deep‐eutectic polymer electrolyte (p‐DEPE) developed here effectively addresses these challenges. As shown in Figure S1a, at the same Li^+^ concentration, with the LiTFSI+LiDFOB+BA system, intermolecular hydrogen bonding between LiTFSI and ECA in the LiTFSI+LiDFOB+ECA system causes electron cloud migration of the central N atom in TFSI^−^ anions toward hydrogen atoms, resulting in a redshift [40]. When BA is introduced into the LiTFSI+LiDFOB+ECA system to adjust the ECA ratio (forming the LiTFSI+LiDFOB+ECA+BA system), a blueshift is observed. This blueshift reflects a weakening of the hydrogen‐bonding interaction between ECA and LiTFSI due to the dilution effect and competitive coordination from BA [41, 42].

Similarly, decreasing the LiTFSI content by introducing LiDFOB into the LiTFSI+LiDFOB+ECA system also causes a blueshift, further confirming that the hydrogen‐bonding strength is modulated by the dual‐salt composition. Importantly, even in the final LiTFSI+LiDFOB+ECA+BA system, a redshift is still observed compared to the LiTFSI+LiDFOB+BA system (without ECA), demonstrating that the ECA–LiTFSI hydrogen bonds persist and dominate the coordination environment.

By establishing a competitive coordination environment through anion‐coupling and intermolecular hydrogen bonding, p‐DEPE mitigates the migration of high‐valent Ni^4^^+^, thereby inhibiting the growth of a resistive Ni^2+^O layer and suppressing undesirable phase transitions. As evidenced in Figure 2b, the bulk layered structure remains largely preserved with p‐DEPE, and the surface rock‐salt phase is significantly thinner compared to PEO‐based samples.

Scanning electron microscopy (SEM) further corroborates the differential impact of the two electrolytes on cathode structural integrity. NCM811 particles cycled with p‐DEPE retain a more coherent morphology after 15 cycles, whereas those with PEO show pronounced cracking (Figure 2c,d). This contrast becomes more evident with extended cycling (25 and 35 cycles), where PEO‐based electrodes exhibit progressive crack propagation driven by persistent interfacial side reactions. Although PEO enables limited short‐term stability at high voltage, accumulating structural damage ultimately compromises long‐term cycling performance. These observations underscore that weakening cathode–electrolyte interaction and controlling nickel valence evolution are critical for achieving stable high‐voltage Li metal batteries. Adsorption energy calculations provide further insight into the interfacial mechanism. As shown in Figure 2f,g, and Figure S2, p‐DEPE exhibits a substantially lower adsorption energy (−10.0 eV) on the cathode surface than PEO (−15.0 eV), indicating that the hydrogen‐bond network formed between salt anions and electronegative polymer groups effectively reduces the solvating power toward high‐valent nickel (PEO based samples: constructing a solvation cluster comprising a PEO chain segment, TFSI^−^ anions, DFOB^−^ anion, and Li^+^ cations. p‐DEPE: constructing a solvation cluster containing BA and ECA monomer units, TFSI^−^, DFOB^−^, and Li^+^, with the hydrogen bonding network included). This competitive intermolecular hydrogen‐bonding strategy thus alleviates severe interfacial side reactions.

To probe the evolution of nickel content and oxidation state at the cathode surface, X‐ray photoelectron spectroscopy (XPS) was performed on Li||NCM811 cells after 25 cycles with varying Ar^+^ sputtering times (Figure 2e and Figure S3). The Ni 2p spectra were deconvoluted to determine the relative contents of Ni^2+^ and Ni^3^^+^. As shown in Figure S3, for p‐DEPE electrolyte, as the etching depth increases, the content of Ni^2+^ of p‐DEPE decreases significantly from 23% to 15%, while the content of Ni^3+^ continuously increases from 42% to 50%. This clear trend demonstrates that the competitive hydrogen bonding network effectively suppresses the reduction of high valent nickel and mitigates the accumulation of low valent Ni^2^^+^O resistive layer. For the Ni‐rich NCM cathodes, the higher Ni content brings more severe cation mixing between Ni and Li ions during cycling because of the similar size of Ni^2+^ (0.7 Å) and Li^+^ (0.8 Å) ions [43]. And the irreversible intermixing easily induces a highly resistant layer on the surface, increasing the risk of uncontrollable structure collapse [44, 45]. In contrast, for PEO electrolyte, the Ni^2+^ and Ni^3+^ contents remain nearly unchanged across the same sputtering depth, indicating that PEO provides no measurable protection against nickel reduction or cation mixing.

Synchrotron‐based soft X‐ray absorption spectroscopy (XAS) offers additional evidence for the local structural and valence changes (Figure 2h). Nickel L‐edge spectra have an L_3_ (2p_3/2 _→ 3d) and an L2 (2p_1/2_ → 3d) region as the result of spin–orbital coupling. The shape, energy, position, and peak intensity ratio contain information on sample valence states [46]. In the normalized Ni L‐edge spectra, PEO‐based samples exhibit a shift of both L_2_ and L_3_ edges to lower energies, consistent with the reduction of high‐valent nickel and phase transformation from layered to spinel/rock‐salt structures [47, 48]. In addition, the intensity ratio between the high‐energy shoulder and the low‐energy shoulder of Ni L3‐edge, which is sensitive to nickel valence, shows a consistent shift toward higher energy for p‐DEPE, confirming less reduction of Ni^4+^/Ni^3+^ [49]. Since the resistive Ni^2^^+^O layer exhibits poor Li^+^ conductivity, its suppression in p‐DEPE is crucial for maintaining rapid ion transport and high‐rate cycling stability.

### 2.2 Suppressing Oxygen Release via Interface Design

Beyond capacity fade, the irreversible release of oxygen from high‐voltage nickel‐rich cathodes poses a critical safety hazard in lithium metal batteries. This process not only accelerates the transformation of the layered structure into disordered and defective rock‐salt phases but also fuels parasitic gas‐evolving reactions. Therefore, stabilizing the cathode structure necessitates a fundamental strategy to mitigate oxygen release by rationally tailoring the cathode‐electrolyte interface.

**FIGURE 3:**
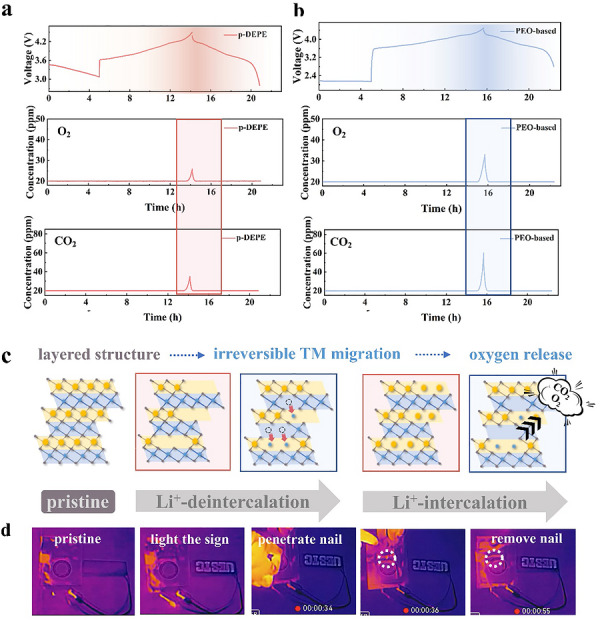
The in situ DEMS spectra with (a) p‐DEPE samples and (b) PEO‐based samples. (c) Conceptual schematic diagram of p‐DEPE and PEO‐based samples in oxygen release. (d) The thermal images of the nail puncture test about p‐DEPE pouch cell.

The in situ differential electrochemical mass spectrometry (DEMS) was employed to probe oxygen evolution during battery operation in the voltage range of 2.8–4.5 V (Figure 3a,b). As shown in Figure 3a, significant O_2_ release is detected upon charging to 4.5 V in cells with PEO‐based electrolyte, coinciding with the H2→H3 phase transition of NCM811 [50]. During the charging process, as electrons are extracted from the lattice, transition metal (TM) ions undergo oxidation. Lattice oxygen in high‐nickel cathodes is increasingly involved in charge compensation under high voltage, rendering lattice oxygen highly unstable due to electron transfer (Figure 3c). This leads to the generation and accumulation of oxygen vacancies, which eventually evolve into O_2_ molecules trapped in transition metal vacancies [51]. Furthermore, the formation of oxygen vacancies further aggravates Li–Ni mixing and undermines lattice stability [52].

In contrast, cells employing p‐DEPE exhibit markedly suppressed O_2_ emission (Figure 3b), confirming that alleviating Li–Ni mixing on the cathode surface is helpful to prevent Ni–Li anti‐sites from acting as nucleation sites for intragranular cracks, which would otherwise cause lattice distortion and crack formation [53]. Moreover, the disordered arrangement of Li^+^ and Ni^2+^/Ni^3+^ ions in the crystal lattice induced by Li–Ni mixing not only blocks the normal transport pathways of lithium ions but also increases the activation energy for Li^+^ diffusion. Therefore, inhibiting the migration of high‐valence nickel ions represents an effective strategy to mitigate structural disorder and detrimental oxygen vacancy formation caused by cation mixing.

Furthermore, p‐DEPE also yields lower CO_2_ signals, indicating suppressed oxidative decomposition of electrolyte components and reduced chemical activity of lattice oxygen at high potentials. Notably, no H_2_ was detected in either polymer system (Figure S4), underscoring a key safety advantage over conventional liquid electrolytes, where proton‐induced cathode corrosion remains a major failure mode [54, 55].

Achieving both safety and stability requires electrolytes that combine interfacial compatibility with intrinsic oxidation resistance. Linear sweep voltammetry (LSV) of Li/Stainless steel (Li||SS) cells reveals that p‐DEPE remains electrochemically stable up to 5.2 V, whereas PEO‐based electrolyte begins decomposing sharply above 4.5 V (Figure S5). This superior anodic stability not only prevents bulk electrolyte oxidation but also promotes the formation of a uniform and robust cathode‐electrolyte interphase (CEI), which further shields the cathode from degradation. And the BA‐based samples reach 4.9 V, which is lower than p‐DEPE. This demonstrates that, in hydrogen‐bond‐dominated systems, the presence of intermolecular hydrogen bonds plays a positive role in enhancing the overall oxidative stability of the system.

To validate the practical safety benefits of this interface design, we conducted nail penetration and cutting tests on cycled pouch cells (Figure 3d and Figure S5). In conventional ether‐based liquid electrolytes, nail penetration typically triggers internal short circuits, leading to gas release, thermal runaway, and cell failure [56]. Remarkably, p‐DEPE‐based pouch cells withstand nail penetration without shorting or combustion. In addition, the connected LED remains illuminated throughout the test (Figure 3d). Infrared thermal imaging shows only localized heating at the puncture site, with no significant temperature rise or cell swelling after nail removal (Figure 3d, Figure S8). Similarly, cutting tests induce neither short circuits nor notable heat accumulation, and the cells continue to function normally post‐test (Figure S8). The same exceptional safety performance is replicated in roll‐type cells (Figures S9 and S10), attributable to the self‐extinguishing character and interfacial stability of p‐DEPE.

### 2.3 Weakening Interfacial Interaction through Coordinative Regulation

The p‐DEPE electrolyte achieves superior interfacial stability by leveraging intermolecular hydrogen bonding to remodel the local coordination environment. This design not only enhances oxidation resistance but also facilitates efficient ion transport, which is critical for high‐rate operation under high voltage. To unravel the underlying mechanism, we conducted molecular dynamics (MD) simulations to analyze the solvation structure and ion coordination.

**FIGURE 4:**
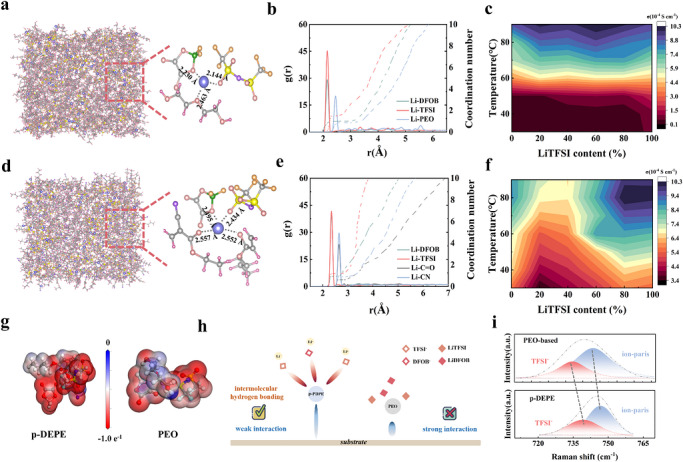
(a) Snapshots obtained from MD simulations of the PEO‐based samples and (b) the corresponding radial distribution functions. (c) Li ions conductivity of c) PEO‐based samples and (f) p‐PDPE samples from 30°C to 90°C. (d) Snapshots of the p‐DEPE samples and (e) Radial distribution functions. (g) Electrostatic potential distribution of the PEO‐based samples and p‐PDPE samples. (h) Conceptual schematic diagram of p‐DEPE and PEO‐based samples in achieving the high Li ions transport through a competitive coordination environment. (i) Raman spectra of p‐DEPE and PEO‐based electrolytes.

In conventional PEO‐based electrolytes, Li^+^ is primarily coordinated by ether oxygen (EO) groups from the polymer backbone, alongside TFSI^−^ and DFOB^−^ anions, forming a tightly bound solvation sheath that restricts Li^+^ mobility (Figure 4a). Radial distribution function (RDF) analysis confirms a high coordination number (CN) for Li^+^ with EO, TFSI^−^, and DFOB^−^, indicating strong ion‐dipole and ion–anion interactions (Figure 4b). In contrast, the solvation structure in p‐DEPE is altered due to intermolecular hydrogen bonding, which weakens the strong coordination effects between Li and polar functional groups and promotes the formation of a fluorinated, cathode‐compatible interphase (CEI) (Figure 4d and Figure S10). In the PEO+LiTFSI+LiDFOB (PEO‐double salt) system, although the double salts are expected to decompose during the cycle to form LiF, which is beneficial for CEI. However, the positive effect of the double salts system on fabricating the stable CEI in the NCM811 system is limited without the intermolecular hydrogen bonding. As shown in Figure S13, in the PEO‐dual‐salt system without intermolecular hydrogen bonding, the unstable PEO‐based samples actually generated more organic fluorides rather than the desired LiF. In BA‐based samples, where intermolecular hydrogen bonding is weaker, a higher LiF content was detected compared to PEO‐based samples. This is attributed to the BA polymer's superior anode stability and the absence of byproduct formation. However, the formation of some organic fluorides is still detected in the F1s spectrum, which may explain why the LiF yield in BA‐based samples was lower than that in p‐DEPE (Figure S12). The highest LiF content of p‐DEPE demonstrates the positive effect of intermolecular hydrogen bonding on promoting the fabrication of LiF‐riched CEI and maximizing the advantages of the double‐salt system.

In p‐DEPE, the labeled distances are 2.463 Å (Li–O (ether oxygen)), 2.144 Å (Li–TFSI^−^), and 2.230 Å (Li–DFOB^−^) (Figure 4a). These short distances indicate strong coordination and restricted Li^+^ mobility [57]. In contrast, Figure 4d presents the coordination environment in p‐DEPE. The distances are 2.557 Å (Li─C═O (ECA)), 2.552 Å (Li─C═O (BA)), 2.434 Å (Li–TFSI^−^), and 2.495 Å (Li–DFOB^−^). The longer distances demonstrate that the competitive hydrogen‐bonding network further demonstrates that the strong confinement of Li^+^ has been alleviated, which helps enhance the diffusion rate of Li ions [17] (Figure 4e). Radial distribution function (RDF) analysis confirms that Li^+^ exhibits high coordination numbers (CN) with EO, TFSI^−^, and DFOB^−^, indicating strong ion‐dipole and ion‐anion interactions. In contrast, the solvation structure in p‐DEPE is altered due to intermolecular hydrogen bonding, which weakens the strong coordination effects between Li and polar functional groups.

Due to the formation of a competitive coordination environment caused by the intermolecular hydrogen bonding in electrolytes, the dissociation energy of Li from the coordination structure declines compared to the PEO‐based samples, which is closely connected to the Li ions transfer [58] (Figure S14). This looser solvation structure lowers the energy barrier for Li^+^ desolvation, thereby accelerating ion transfer kinetics. This conclusion is supported by a higher Li^+^ transference number (0.6) in p‐DEPE compared to PEO (Figure S12). Besides, even with an additional 20% lithium salt content, the Li^+^ transference number achievable in the BA dual‐salt system cannot exceed that of p‐DEPE at 1.125 m (Figure S15b,c).

Ionic conductivity studies across temperatures (30–90°C) and salt compositions further highlight the role of optimized coordination. While the conductivity of PEO is largely temperature‐dependent and insensitive to salt ratio (Figure 4c), p‐DEPE exhibits a strong composition–performance relationship. At LiTFSI contents ranging from 20% to 40%, the ionic conductivity of samples using dual salts (LiTFSI/LiDFOB) is lower than that of single‐salt samples across all temperatures. Dual‐salt systems outperform single‐salt electrolyte only at high LiTFSI content (90%), achieving a room‐temperature conductivity of 5.7 × 10^−4^ S cm^−1^ (Figure 4f). This non‐linear enhancement underscores the synergistic effect of competitive hydrogen bonding and dipole interactions in facilitating Li^+^ transport. The ionic conductivity of the BA+dual‐salt system (weak hydrogen bonding) and the p‐DEPE system (strong hydrogen bonding) at the same total lithium salt concentration (1.125 M), while varying the LiTFSI: LiDFOB ratio are shown in Figure S16. For p‐DEPE electrolyte, the ionic conductivity is highly sensitive to LiTFSI content. As the LiTFSI proportion increases, the enhanced hydrogen bonding (more TFSI^−^ available to interact with ECA) reduces the distance between Li^+^ and polar functional groups, weakening the constraint on Li^+^ and leading to higher conductivity. For BA+dual‐salt electrolyte (weak hydrogen bonding), over the same range of LiTFSI content, the ionic conductivity shows little variation. This demonstrates that simply increasing the concentration of TFSI^−^ anions (and thus their participation in the solvation structure) does not significantly improve conductivity in the absence of strong hydrogen bonding.

To confirm the p‐DEPE qualifies as a deep eutectic polymer electrolyte (DEPE), we performed differential scanning calorimetry (DSC) on four samples. The base polymer (ECA+BA), the polymer with LiTFSI, the polymer with LiDFOB, and the polymer with dual salts (LiTFSI+LiDFOB). As shown in Figure S17, the pure polymer (ECA+BA) exhibits a melting point above 80°C. Upon addition of LiTFSI or LiDFOB alone, the melting point drops to approximately 40°C. The dual‐salt system (p‐DEPE) shows the lowest melting point, lower than that of any single component, satisfying the defining criterion of a deep eutectic system (a melting point depression below the ideal eutectic temperature). This result demonstrates that our electrolyte is a deep eutectic polymer, where intermolecular hydrogen bonding between lithium salts and polymerizable monomers (ECA, BA) leads to a significant depression of the melting point. And narrowing the DSC temperature range from −80°C to 0°C can clearly reveal that the dual‐salt configuration significantly lowers the glass transition temperature (T_g_) of p‐DEPE from −80°C to 0°C (Figure S18), indicating enhanced polymer chain mobility in the amorphous phase. This reduction in crystallinity facilitates segmental motion and promotes faster Li^+^ conduction.

Electrostatic potential (ESP) mapping illustrates a lower electron density at the p‐DEPE–cathode interface relative to PEO (Figure 4g), confirming the weakened adhesive interaction between the electrolyte and NCM811 surface [59]. This interfacial decoupling is crucial for minimizing parasitic reactions and enabling stable high‐voltage cycling (Figure 4h). Raman spectroscopy provides further evidence of the altered solvation environment. In p‐DEPE, characteristic TFSI^−^ peaks (743 cm^−1^ for ion pairs, 737 cm^−1^ for free anions) exhibit a blue shift [60, 61, 62], indicating increased anion participation in the solvation shell and greater LiTFSI dissociation (Figure 4i) [63, 64, 65]. These changes promote a higher population of free Li^+^, as corroborated by the upfield shift in ^7^Li NMR spectra (Figure S21). These multimodal analyses demonstrate that hydrogen‐bond‐mediated coordination structure of p‐DEPE electrolyte simultaneously weakens deleterious cathode–electrolyte interactions and enhances Li^+^ transport.

### 2.4 Protecting NCM811 Structural Integrity with p‐DEPE Electrolyte

To unravel the complex interfacial evolution in Li||NCM811 batteries, distribution of relaxation times (DRT) analysis was applied during the first cycle (2.8–4.5 V), effectively decouples individual electrochemical processes based on their characteristic timescales [66]. In PEO‐based cells, the charge transfer resistance (τ_3_, 10^−3^–10^−1^ s) dominates the impedance spectrum, indicating a high energy barrier for interfacial charge transfer. This significant resistance impedes Li^+^ diffusion and reflects substantial interfacial incompatibility. In stark contrast, p‐DEPE cells exhibit markedly lower and more reversible τ_3_ peaks, signifying mitigated charge transfer resistance and suppressed cation mixing within the NCM811 lattice. Furthermore, the Li^+^ diffusion process (τ_4_, ∼10 s) is more pronounced in p‐DEPE, highlighting enhanced bulk ionic transport.

**FIGURE 5:**
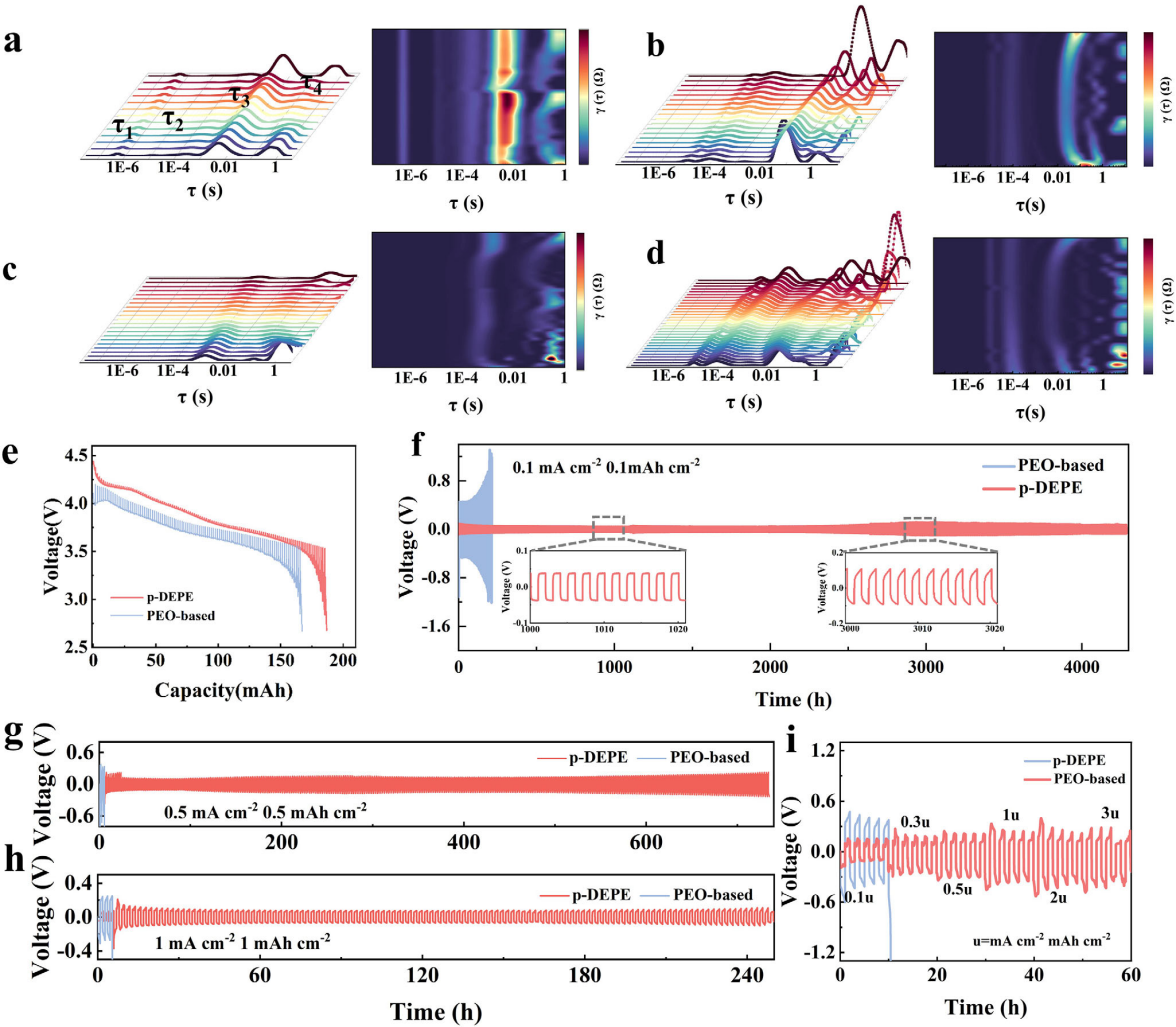
The DRT spectra of (a) PEO‐based c) p‐DEPE samples at room temperature. The DRT spectra of (b) PEO‐based (d) p‐DEPE samples at 70°C. (e) GITT discharge profiles of NCM811 cathodes with p‐DEPE samples and PEO‐based samples. The Li symmetric cells with p‐DEPE and PEO‐based samples cycled at (f) 0.1 mA cm^−2^ and 0.1 mAh cm^−2^, (g) 0.5 mA cm^−2^ and 0.5 mAh cm^−2^, and (h) 1 mA cm^−2^ and 1 mAh cm^−2^. (i) The rate performance of Li symmetric cells with p‐DEPE and PEO‐based samples.

In PEO‐based cells (Figure 5a), the τ_1_ at 10^−6^ s represents the contact resistance, corresponding to the physical contact at the electrode/electrolyte interface. τ_2_ peak (10^−5^ to 10^−3^ s) is associated with the ion transport through the interfacial CEI and SEI layers. The charge transfer resistance (τ_3_, 10^−3^–10^−1^ s) dominates the impedance spectrum, indicating a high energy barrier for interfacial charge transfer. This significant resistance impedes Li^+^ diffusion and reflects substantial interfacial incompatibility [67]. In stark contrast, p‐DEPE cells exhibit markedly lower and more reversible τ_3_ peaks(Figure 5c), signifying mitigated charge transfer resistance and suppressed cation mixing and Li deficiency within the NCM811 lattice [68]. Furthermore, the Li^+^ diffusion process (τ_4_, ∼10 s) is more pronounced in p‐DEPE, highlighting enhanced bulk ionic transport.

For the BA+LiTFSI+LiDFOB system (BA‐based samples), where intermolecular hydrogen bonding is weak but the double salt remains effective. As shown in Figure S20, due to the in situ polymerization of BA‐based samples, the τ_1_ peak at 10^−6^ s indicates that the contact resistance cannot be observed. This confirms that the BA‐based samples possess good physical contact at the electrode/electrolyte interface. However, τ_3_ (10^−3^–10^−1^ s), representing the charge transfer resistance, still dominates the BA‐based impedance spectrum, indicating a high energy barrier for interfacial charge transfer. This high‐resistance interface significantly impedes Li^+^ diffusion caused by the poor interfacial compatibility between the BA‐based electrolyte and the cathodes [67]. In contrast, in the p‐DEPE system, where intermolecular hydrogen bonding plays a dominant role, the lower charge transfer resistance demonstrates the p‐DEPE has excellent compatibility with NCM cathodes and excellent interfacial ion transport kinetics.

At elevated temperature (70°C), the DRT profiles further demonstrate the stability advantage of p‐DEPE. While the softened PEO electrolyte adheres excessively to the cathode and causes the disappearance of the contact resistance peak τ_1_ (Figure 5b and Figure S19), it fails to improve charge transfer kinetics. Conversely, the τ_3_ peak of p‐DEPE shifts to a lower position (Figure 5d), confirming accelerated Li^+^ diffusion. The persistent τ_1_ peak in p‐DEPE also indicates maintained and favorable electrode‐electrolyte contact, aided by the adhesive hydrogen‐bonding network [69]. GITT analysis quantitatively supports these findings. The calculated Li^+^ diffusion coefficient (D _Li+_) is significantly higher in p‐DEPE‐based cells, especially during the initial charging phase (Figure 5e). This rapid ionic replenishment at the interface is a cornerstone for stable high‐rate cycling, demonstrating that the hydrogen‐bond‐modulated interface not only protects the cathode structurally but also facilitates superior ionic kinetics.

Symmetrical Li||Li batteries were assembled with various electrolytes to test the lithium metal anode compatibility. The PEO‐based cells exhibit obviously increased overpotential over 200 h at 0.1 mA cm^−2^ (0.1 mAh cm^−2^), as shown in Figure 5f. Li||Li cells with p‐DEPE demonstrate remarkably stable plating/stripping over 4000 h with a low overpotential (∼50 mV), far exceeding the performance of PEO‐based cells. This stability extends to higher current densities. P‐DEPE cells cycle stably for over 700 h at 0.5 mA cm^−2^ (Figure 5g) and over 250 h at 1 mA cm^−2^ (Figure 5h), whereas PEO cells fail rapidly above 0.1 mA cm^−2^. Rate capability tests further reveal that p‐DEPE cells can operate at 3 mA cm^−2^ with a manageable overpotential (∼500 mV), highlighting exceptional Li plating/stripping reversibility (Figure 5i). This performance is attributed to the anion‐enriched electric double layer promoted by the unique solvation structure, which guides uniform Li deposition [70] Post‐cycling SEM confirms a dense and dendrite‐free Li morphology in p‐DEPE cells, in contrast to the irregular, dendritic growth observed with PEO (Figure S22).

### 2.5 Achieving Stable High‐Voltage Electrochemical Performance

**FIGURE 6:**
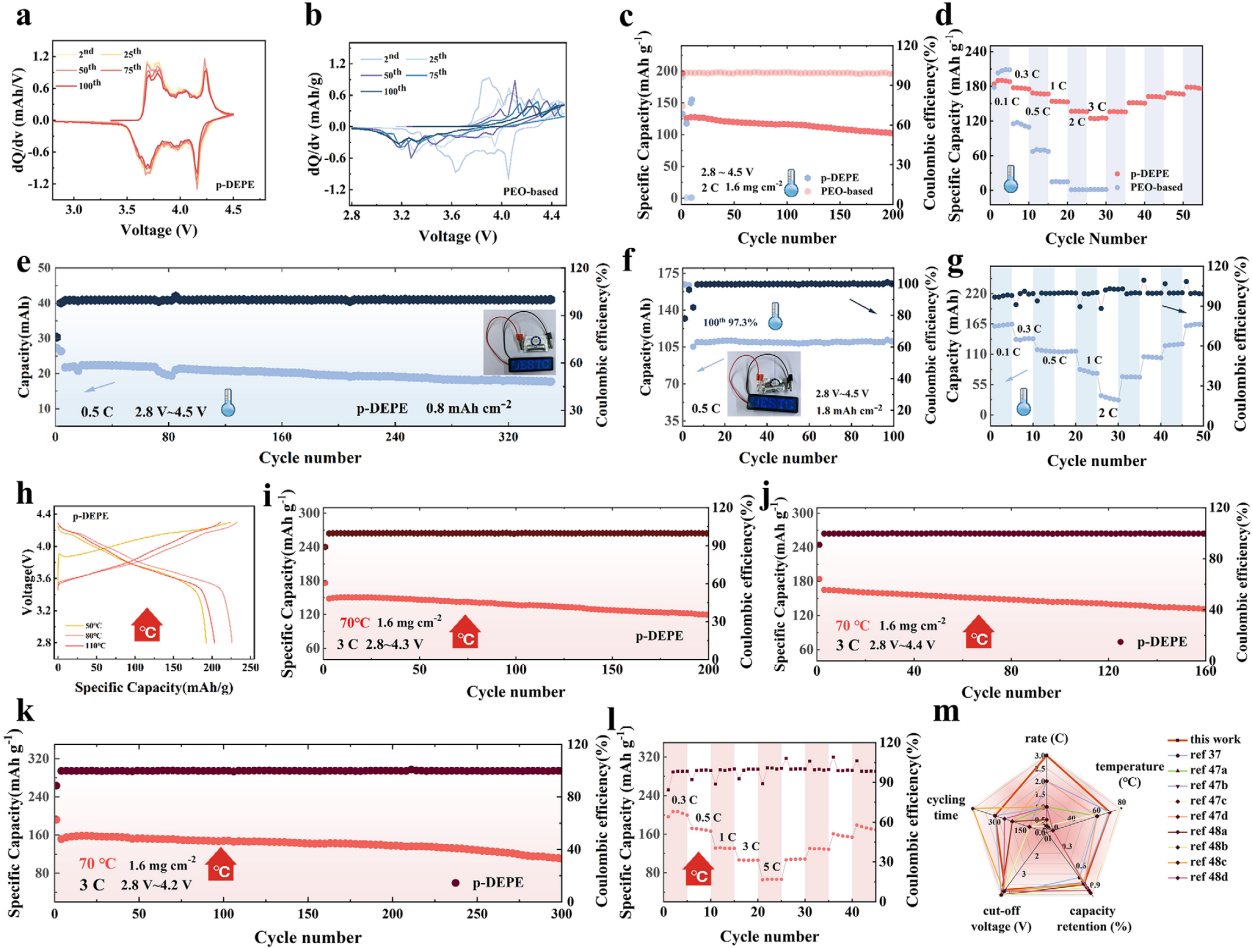
Galvanostatic charge/discharge profiles of NCM811 at cut‐off voltages of 4.5 V versus Li^+^/Li with (a) p‐DEPE samples and (b) PEO‐based samples. (c) The Li||NCM811 batteries with p‐DEPE samples and PEO‐based samples cycled at 2 C. (d) The rate performance of Li||NCM811 batteries with p‐DEPE samples and PEO‐based samples. The Li||NCM811 pouch roll batteries with p‐DEPE samples cycled at 0.5 C under (e) 0.8 mAh cm^−2^ and (f) 1.8 mAh cm^−2^. (g) The rate performance of Li||NCM811 pouch roll batteries with p‐DEPE samples. (h) The Li||NCM811 batteries with p‐DEPE samples were cycled at different temperatures. The Li||NCM811 batteries with p‐DEPE samples cycled at 3 C under 70°C with the cut‐off voltage at (i) 4.3 V, (j) 4.4 V, and (k) 4.2 V. (l) The rate performance of Li||NCM811 coin batteries with p‐DEPE samples at 70°C. (m) Comparison of the cycling performance of Li||NCM811 cell in this study with other reported data under extreme conditions, such as high temperature and high rate.

The validation of a high‐voltage electrolyte lies in its ability to sustain the structural and electrochemical integrity of Ni‐rich cathodes under cycling. Differential capacity (dQ/dV) analysis provides deeper insight into these contrasting behaviors (Figure 6a,b). The characteristic redox peaks corresponding to the sequential phase transitions of NCM811 (H1→M→H2→H3) [14] are well‐preserved in p‐DEPE cells throughout cycling. Conversely, in PEO cells, the H2–H3 peak vanishes by the 5th cycle, indicating irreversible structural damage and active material loss [25]. This degradation is attributed to the large anisotropic lattice strain during the H2–H3 transition, which promotes microcrack formation and impedes redox kinetics when coupled with a reactive interface. Cyclic voltammetry further corroborates this finding, as shown in Figure S23 PEO‐based cathodes show pronounced peak decay at ∼4.2 V after 25 cycles, while p‐DEPE cathodes maintain strong, stable redox activity.

The exceptional interfacial compatibility and electrochemical stability of p‐DEPE translate to outstanding high‐rate performance. The p‐DEPE batteries demonstrate a high discharge capacity of 192 mAh g^−1^ in the initial active cycle, which benefits from lower charge transfer resistance (Figure S24). When the cycling rate up to 2C, p‐DEPE cell retains 80% of their capacity after 200 cycles at 4.5 V, sustaining a reversible capacity of 110 mAh g^−1^, significantly outperforming the BA double‐salt system (Figure 6c and Figure S26). PEO cells, in stark contrast, fail rapidly under the same conditions. Rate capability tests reveal that p‐DEPE cells can deliver 189, 177, 167, 153, 136, and 124 mAh g^−1^ at 0.1, 0.3, 0.5, 1, 2, and 3 C, respectively, with near‐perfect capacity recovery upon returning to 0.1 C (Figure 6d).

To assess practical viability, we fabricated high‐loading pouch cells. A cell with the areal capacity of 0.8 mAh cm^−2^ retains 80% capacity after 320 cycles at 0.5 C in the voltage range of 2.8–4.5 V (Figure 6e). The corresponding charge–discharge voltage profiles at different cycles are shown in Figure S25. More impressively, a cell with a high areal loading of 1.8 mAh cm^−2^ maintains 98.3% capacity after 100 cycles at 4.5 V (Figure 6f), demonstrating exceptional interfacial stability under conditions relevant to commercial applications. Furthermore, these pouch cells exhibit remarkable safety, maintaining function during nail penetration and cutting tests without thermal runaway (inset, Figure 6e). Performance remains robust even at elevated cut‐off voltages of 4.6, 4.7, and 4.8 V, confirming the extended voltage window enabled by p‐DEPE electrolyte (Figure S27). Additionally, roll batteries based on p‐DEPE electrolyte show a superior rate performance (Figure 6g), delivering specific capacities of 164 mAh g^−1^ at 0.1 C. Surprisingly, when the current density turns to 0.1 C, the specific capacities is close to the original ones (162 mAh g^−1^), indicating the good reversibility of p‐DEPE cells.

The electrolyte also demonstrates outstanding thermal resilience. Cells operate stably from 50°C to 90°C (Figure 6h). At 70°C and 3 C, p‐DEPE cells achieve 200 cycles with 80% capacity retention, a feat unattainable by PEO cells (Figure 6i). This stability persists at high voltage. At 4.4 V and 70°C, p‐DEPE cells complete over 160 cycles with 80% retention (Figure 6j), and at 4.2 V, cycling stability is further enhanced (Figure 6k). High‐temperature rate performance is also superior, with capacities of 206, 172, 131, 106, and 66 mAh g^−^^1^ at 0.3, 0.5, 1, 3, and 5 C, respectively, and 87% capacity recovery upon returning to 0.3 C (Figure 6l). As summarized in Figure 6m and p‐DEPE achieve a rare combination of high cut‐off voltage (> 4.5 V), high operational temperature (up to 90°C), excellent rate capability (up to 5 C), and long‐term cycling stability, outperforming most advanced polymer‐based electrolytes reported for Ni‐rich NCM811 cathodes [71, 72, 73, 74].

At high temperatures, oxygen release becomes more severe due to lattice distortion and oxygen vacancy enrichment caused by lithium‐nickel mixing on the cathode surface, having stricter requirements on the stability of NCM811 batteries. Because the migration of nickel from the cathode surface into the polymer in p‐DEPE has been suppressed, which is beneficial to reduce the formation of low‐valent, high‐resistance nickel oxide, and promotes the formation of a LiF‐rich CEI layer. The enhanced interfacial stability and ion transport kinetics of p‐DEPE enable the outstanding electrochemical performance at 70°C 3C, which can maintain the stable cycling for more than 300 cycles with 80% capacity retention. As summarized in Figure 6m, the p‐DEPE electrolyte outperforms most previously reported polymer‐based electrolytes for Ni‐rich NCM cathodes in terms of cycling stability, rate capability, operating temperature, and cut‐off voltage, highlighting the advantage of the competitive hydrogen bonding design [57, 71, 72, 73, 74, 75, 76, 77, 78]. (Table S1).

## 3 Conclusions

In summary, we have developed a polyacrylate‐based deep‐eutectic polymer electrolyte that effectively stabilizes the interface between Li metal anodes and high‐voltage Ni‐rich NCM811 cathodes. The key to this stability lies in a competitive hydrogen‐bonding coordination network, which shields electronegative functional groups and reduces their adverse solvation of high‐valent nickel species. This mechanism simultaneously suppresses cathode‐electrolyte interfacial degradation, mitigates oxygen release, and retards the layered‐to‐rock‐salt phase transition, while ensuring high oxidative stability (> 5.2 V) and ionic conductivity (0.6 mS cm^−^^1^). As a consequence, the Li||NCM811 batteries maintain stable cycling with 80% capacity retention after 300 cycles at 3 C under 70°C. This work provides a new polymer strategy for promoting the improvement of high‐voltage Li metal batteries under high‐rate operations.

## Author Contributions

**Dongjiang Chen**: conceptualization, writing – original draft, funding acquisition, writing – review and editing, project administration, validation. **Wei Chen**: conceptualization, methodology. **Yuxin Fan**: conceptualization, investigation, funding acquisition, validation, writing – original draft. **Yichao Yan**: validation, project administration, funding acquisition. **Yin Hu**: software, formal analysis. **Tianyu Lei**: validation, visualization, writing – review and editing. **Miao He**: investigation, methodology, validation.

## Conflicts of Interest

The authors declare no conflicts of interest.

## Supporting information




**Supporting File 1**: advs75883‐sup‐0001‐SuppMat.docx.

**Supporting File 2**: advs75883‐sup‐0002‐VideoS1.mp4.

**Supporting File 3**: advs75883‐sup‐0003‐VideoS2.mp4.

**Supporting File 4**: advs75883‐sup‐0004‐VideoS3.mp4.

## Data Availability

The data that support the findings of this study are available from the corresponding author upon reasonable request.
