# Supplementary material for: Deep Eutectic Polymer Electrolyte with Competitive Hydrogen‐Bonding Coordination for High‐Voltage Nickel‐rich Lithium Metal Batteries
Source: Adv Sci (Weinh). 2026 May 30;13(47):e75883. doi: 10.1002/advs.75883 (PMC13336130; doi:10.1002/advs.75883)
Supplement: Supplementary file 1 [file ADVS-13-e75883-s001.docx]

**Supporting Information**

**Deep Eutectic Polymer Electrolyte with Competitive Hydrogen-Bonding Coordination for High-Voltage Nickel-rich Lithium Metal Batteries**

*Yuxin Fan, Miao He,* *Yin Hu, Wei Chen, Yichao Yan*, Tianyu Lei*, Dongjiang Chen**

*Y. Fan, M. He, Y. Hu, W. Chen, Y. Yan, T. Lei, D. Chen*

State Key Laboratory of Electronic Thin Films and Integrated Devices, University of Electronic Science and Technology of China, Chengdu 610054, China.

Email: yanyichao@uestc.edu.cn, leity@uestc.edu.cn, dongjiang.chen@uestc.edu.cn

**Materials**

Ethyl cyanoacrylate (ECA, purity >98%), butyl acrylate (BA, purity ≥99%, containing 10–60 ppm MEHQ), polyethylene oxide (PEO, molecular weight approximately 600000), and 2,2-azobis(isobutyronitrile) (AIBN, purity >98%) were purchased from Aladdin Reagent (Shanghai) Co., Ltd. Lithium bis(trifluoromethanesulfonimide) (LiTFSI, purity: 99.9%), lithium difluorooxalate borate (LiDFOB, purity: 99.9%), fluorinated ethylene carbonate (FEC, purity: 99.9%), PVDF (HSV900, molecular weight approximately 100,000), and diethyl carbonate (DEC, purity: 99.99%) were purchased from Suzhou Duoduo Chemical Technology Co., Ltd. NMC811, Celgard 2500 separators, and Super P were purchased from Guangdong Kangruide New Energy Technology Co., Ltd. (China).

**Synthesis**

For p-DEPE electrolyte, the optimal ratio of lithium salts (1.86 g LiTFSI and 0.21 g LiDFOB) was added separately to glass beakers containing 1.2 mL of BA monomer and 0.8 mL of ECA monomer, respectively, and stirred for 1 hour until the liquid became transparent. Subsequently, the BA monomer and ECA monomer were added to a glass beaker containing 4 mL of DEC, 2 mL of FEC, and 2 wt% AIBN to prepare the p-DEPE precursor. This precursor was applied to the surfaces of lithium metal and the cathode via dip-coating to assemble the battery; the assembled battery was then heated and polymerized in an oven at 60 °C for 10 hours.

For PEO-based electrolyte, place 0.5 g of LiTFSI, 0.063 g of LiDFOB, and 1.6 g of PEO into a glass bottle, and add 14 mL of acetonitrile, and stir for 24 hours. Then, spin-coat the polymer onto a polytetrafluoroethylene (PTFE) template. The spin-coated PEO film was placed in a vacuum oven and dried at 60 °C for 24 hours, then cut into pieces to obtain the final PEO polymer electrolyte.

**Characterizations**

The physical morphology was characterized by scanning electron microscopy and Transmission Electron Microscope (SEM, NanoSEM 450, FEI and TEM, FEI Tecnai G2 F20). X-ray photoelectron spectroscopy (XPS, Thermo Fisher Scientific Nexsa G2) was employed to analyze the element and surface chemistry of the samples. Raman spectrum (Quantum-I Plus 600 MHz, 532 nm, 600-2000 cm^−1^) was used to observe the variation of TFSI^−^ in the electrolyte. Differential scanning calorimetry (HITACHI DSC200, −80 ℃ - 0 ℃) was used to test the glass transition temperature (T_g_). NMR spectra were recorded on a Quantum-I Plus 600 MHz NMR spectrometer at room temperature. The assembled unactivated Li/NCM811 battery was charged/discharged at 0.1C for 30 minutes and then subjected to an impedance test once until the charge/discharge cut-off voltage (4.5V/2.8V) was reached for DRT tests. For in-situ DEMS, the cell is charged/discharged at 0.1 C between 2.8 and 4.5 V. The evolved gases are continuously monitored by a mass spectrometer. For XPS etching test, the Li||NCM811 coin cell is first activated for 2 cycles at 0.1 C (2.8–4.5 V), then cycled for an additional 23 cycles at 0.3 C. After cycling, the cell is disassembled in an Ar‑filled glovebox. The cathode electrode is rinsed with DEC to remove residual electrolyte and then transferred under inert atmosphere to the XPS instrument. Cluster etching was used, and the etching depth was 10 nm. A total of 4 times was etched, corresponding to total depths of approximately 10, 20, 30, and 40 nm. Infrared Thermal Imaging Test: The Li||NCM811 pouch cells (single-layer pouch cells with a surface loading of 4.3 mg/cm^-2^, cathode area: 4 cm × 8 cm)) were assembled for puncture tests and cut tests.

**Electrochemical Measurements**

The cycling and rate performance were measured on the Land Battery Testing System at room temperature. CHI660E electrochemical workstation was employed to collect the electrochemical impedance spectra (EIS) and linear polarization curves.

The Li||NCM811 cells were assembled using CR2025-type coin cells under an argon atmosphere. The NCM811 composite cathode was prepared by mixing NCM811 powder, polyvinylidene fluoride (PVDF), LiTFSI, LiDFOB powder and Super P in a weight ratio of 8:0.65:0.028:0.07:0.65 with N-Methylpyrrolidone (NMP) in Ar-filled glovebox. The above obtained slurry was then coated on Al foil, and dried at 60 ºC overnight in vacuum to finally produce the composite cathode film with a loading of ~ 1.6 mg cm^−2^. Then, 20μL p-PDPE precursor was dripped on top of the NCM811 composite cathode, followed by a piece of supporting separator, 20μL p-PDPE precursor and lithium-metal chip in sequence. To improve the stability of the battery, the Celgard 2500 was used as the supporting separator, and then the whole cell was placed in an oven at 60 ºC for 24 h. The solid cells were then subjected to galvanostatic charge/discharge and EIS measurements. For the roll cells, the cathode area of p-DEPE roll cell is 3 cm × 11.5 cm, and the NCM811 loading is 4.3 mg cm^-2^ with 1.5 ml precursor solution. The N/P ratios are 51.5. And when NCM811 loading up to 9.6 mg cm^-2^, the addition of precursor solution is 1.5 ml, and the N/P ratios are 22.9. The p-DEPE pouch cell used for puncture and cut tests is a single-layer, single-sided pouch cell (NCM811 area: 4 cm × 8 cm), with a loading of 4.3 mg cm^-2^ and addition of 1 ml precursor solution.

**Theoretical calculations**

Using the Dmol3 module of Materials Studio, calculations were performed at the software's default fine precision level based on the GGA-PBE functional. Core treatment: All Electron Basis set: DNP SCF tolerance: 1×10⁻⁶ eV/atom DIIS size: 6. First, structural optimization was performed for each cluster. Based on the optimized structures, further charge analysis was conducted on the molecules to obtain their electrostatic potential distribution information. Subsequently, Li⁺ was removed, and the energy change before and after dissociation was calculated to obtain the Li⁺ dissociation energy E:

$$E=E_{a}+E_{b}{-E}_{ab}$$

where E_ab_ is the pre-dissociation system energy, and E_a_ and E_b_ are the energies of the two post-dissociation components, respectively.

Materials Studio software was employed for structural modeling, and all structural optimization calculations were performed using the open-source CASTEP module. The crystal structure of NCM811 was extracted from public databases. The GGA-PBE functional was adopted throughout the calculations, with parameters set as follows: the "Metal" option was enabled, and both spin-polarized and non-polarized states of the system were considered. The electronic energy cutoff was set to 290 eV, the k-point mesh was configured as 2×2×1, ultrasoft pseudopotentials were utilized, relativistic effects were treated by the Koelling-Harmin scheme, and the self-consistent field (SCF) convergence criterion was set to 2×10^-6^ eV/atom.

The detailed calculation procedure is as follows. First, structural optimization was carried out for the pristine NCM811 crystal. The (001) crystal plane was cleaved from the relaxed NCM811 structure and expanded to an appropriate supercell size. A vacuum layer of 30 Å was introduced along the (001) direction to eliminate interactions induced by periodic boundary conditions. Ionic solvation clusters of two distinct systems were respectively loaded into the expanded NCM811 structure, and their adsorption energies E were calculated according to the formula:

$$E=E_{ab}-E_{a}-E_{b}$$

where E_ab_ represents the total energy of the adsorbed complex system, and E_a_, E_b_ denote the energies of the two isolated components before adsorption, respectively.

MD simulations were performed to study the molecular adsorption behavior of the diffusion of Li, Cl and O atoms. Our simulation calculation was conducted on four structures with an integration time-step of 1 fs. Periodic boundary conditions were applied in the x- and y-dimensions. First, the conjugate gradient algorithm and energy minimization were performed to obtain a stable structure. The Condensed-phased Optimized Molecular Potential for Atomistic Simulation Studies force field was also used to optimize these structures in the Materials Studio with forcite Module. Each sample was then equilibrated under the NPT ensemble at a constant temperature of 300K to achieve an equilibrium state with zero pressure for 10 ns. Furthermore, a potential cutoff radius of 2.25 nm is applied in the calculation of the non-bonded interaction. And the PPPM has been used to describe the electrostatic. The Andersen feedback thermostat and Berendsen barostat algorithm are applied in the system with temperature and pressure conversion. Finally, the properties of our structures are obtained in the last 20 ns.

The Vienna Ab Initio Package (VASP) was employed to perform all the density functional theory (DFT) calculations within the generalized gradient approximation (GGA) using the Perdew, Burke, and Enzerhof (PBE) formulation.^[1-3]^ The projected augmented wave (PAW) potentials were applied to describe the ionic cores and take valence electrons into account using a plane wave basis set with a kinetic energy cutoff of 450 eV.^[4,5]^ Partial occupancies of the Kohn–Sham orbitals were allowed using the Gaussian smearing method and a width of 0.05 eV. The electronic energy was considered self-consistent when the energy change was smaller than 10^−5^ eV. A geometry optimization was considered convergent when the force change was smaller than 0.05 eV/Å. Grimme’s DFT-D3 methodology was used to describe the dispersion interactions.^[6]^ The vacuum spacing perpendicular to the plane of the structure is 20 Å. The Brillouin zone integral utilized the surface structures of 1×1×1 monkhorst pack K-point sampling. Finally, the adsorption energies(Eads) were calculated as Eads= Ead/sub -Ead -Esub, where Ead/sub, Ead, and Esub are the total energies of the optimized adsorbate/substrate system, the adsorbate in the structure, and the clean substrate, respectively.

**Figure S1** a) Raman spectra of the ECA system at the same Li^+^ concentration; b) Raman spectra of the double-salt system at the same Li^+^ concentration.

**Figure S2.** The adsorption energy of a) p-DEPE without dual salts (Eads=-3.015 eV

), b) ECA with dual salts (Eads=-2.385 eV) and c) BA with dual salts (Eads=-2.498 eV) on the surface of NCM811 cathodes

**Figure S3.** Ni 2p XPS etching spectra of (a) p-DEPE and (b) PEO-based samples.

**Figure S4.** The in-situ DEMS spectra with PEO-based samples (a) and p-DEPE (b) about H_2_.

**Figure S5.** The LSV curves of p-DEPE, BA-based and PEO-based samples.

**Figure S6.** The thermal images of cut test about p-DEPE pouch cell.

**Figure S7.** The p-DEPE pouch cell after nail puncture tests with the lit light board.

**Figure S8.** The p-DEPE pouch cell after cut tests with the lit light board.

**Figure S9.** The p-DEPE roll cell after nail puncture tests with the lit light board.

**Figure S10.** The combustion test of p-DEPE.

**Figure S11.** The XPS images of PEO-based samples (a) and p-DEPE (b) after 25 cycles.

**Figure S12.** F1s XPS etching spectra of the a) p-DEPE, b) PEO-based samples and c) BA-based samples.

**Figure S13** C1s XPS etching spectra of the a) p-DEPE, b) PEO-based samples and c) BA-based samples.

**Figure S14.** The binding energy between Li and the typical coordination structure in PEO-based samples and p-DEPE electrolytes.

**Figure S15.** a) The Li ions migration number of p-DEPE. b) Ionic mobility of the BA with double-salt system when the total lithium salt concentration is reduced by 30%; c) Ionic mobility of the BA with double-salt system when the total lithium salt concentration is increased by 20%.

**Figure S16.** Ionic conductivity of the BA with double-salt system and p-DEPE at different LiTFSI contents while maintaining the same total Li^+^.

**Figure S17** DSC curves of polymer (BA+ECA) samples with different Li salts.

**Figure S18.** The DSC curves of p-DEPE with no Li salts, single Li salts and dual Li salts.

**Figure S19.** a) EIS and DRT curves of the PEO-based sample during charging at room temperature; b) EIS and DRT curves of the PEO-based sample during discharging at room temperature; c) EIS and DRT curves of the p-DEPE sample during charging at room temperature; d) EIS and DRT curves of the p-DEPE sample during discharging at room temperature; e) EIS and DRT curves of the PEO-based sample during charging at 70 °C; f) EIS and DRT curves of the PEO-based sample during discharging at 70 °C; g) EIS and DRT curves of the p-DEPE sample during charging at 70 °C; h) EIS and DRT curves of the p-DEPE sample during discharging at 70 °C.

**Figure S20** Charge/discharge DRT curves for the a) PEO-based samples, b) BA-based samples, and c) p-DEPE.

**Figure S21.** The ^7^Li NMR spectra of p-DEPE and PEO-based electrolyte.

**Figure S22.** The SEM images of Li deposition morphology after 20 cycles with PEO-based samples (a) and p-DEPE (b).

**Figure S23.** The CV curves of Li||NCM811 with p-DEPE and PEO-based cells after 25 cycles.

**Figure S24.** The EIS images of Li||NCM811 cells with PEO-based and p-DEPE after 20 cycles.

**Figure S25.** The charge-discharge voltage profiles of PEO-based samples (a)and p-DEPE (b).

**Figure S26.** Cycling performance of Li||NCM811 batteries assembled using the PEO-based samples, BA-based samples, and p-DEPE from 2.8V to 4.5 V at a 2C.

**Figure S27.** The charge-discharge voltage profiles of p-DEPE with different cut-off voltage.

**Supplementary Table 1** | Comparison of cycling performance for previously reported LMBs.

| Modification methods | Cycle number (n) | Temperature (℃) | Rate (C) | Ref. |
| --- | --- | --- | --- | --- |
| BE-DNE | 200  300 | 25  60 | 1  1 | ^[7]^ |
| L-AGPE | 500  100 | 25  70 | 1  2 | ^[8]^ |
| WSGPE | 200  100 | 25  80 | 0.5  0.5 | ^[9]^ |
| FFP-PE | 200  500 | 60  50 | 1  1 | ^[10]^ |
| GPE-F | 200  150 | 60  70 | 2  2 | ^[11]^ |
| PFEA-DEE | 200 | 25 | 0.5 | ^[12]^ |
| s-PE4 | 200 | 25 | 0.5 | ^[13]^ |
| TF+HCE | 431 | 25 | 1 | ^[14]^ |
| CPPE | 245 | 25 | 1 | ^[15]^ |
| p-DEPE | 200  300 | 30  70 | 2C  3C | **This work** |

**Reference**

[1] Kresse, G., Furthmüller, J. Phys. Rev. B 1996, **54**, 11169–11186.

[2] Perdew, J. P., Burke, K., et al., Phys. Rev. Lett. 1996, **77**, 3865–3868.

[3] Kresse, G., Joubert, D. Rev. B 1999, **59**, 1758-1775.

[4] Blöchl, P. E. Phys. Rev. B 1994, **50**, 17953–17979.

[5] Grimme, S., Antony, J., et al., Chem. Phys. 2010, **132**, 154104.

[6] Henkelman, G., Uberuaga, B. P., et al., Chem. Phys. 2000, **113**, 9901.

[7] R. Xu, A. Hu, et al., Angew Chem Int Ed Engl 2025, **64**, e202513321.

[8] H. Yang, J. Yan, et al., Energy & Environmental Science 2025, **18**, 9854.

[9] S. Liu, W. Tian, et al., Nat Commun 2025, **16**, 2474.

[10] L. Xu, M. Li, et al., Chinese Chemical Letters 2026, **37**.

[11]	Z. Peng, J. Lin, et al., Angew Chem Int Ed Engl 2026, e22407.

[12]	J. Li, C. Li, et al., ACS Nano 2026, **20**, 9250.

[13] D. Zhang, T. Tian, et al., Advanced Functional Materials 2025, **36**.

[14]	Y. Lu, Y. Liu, et al., Energy & Environmental Science 2025, **18**, 9512.

[15] H. Wang, S. Duan, et al., J Am Chem Soc 2026.
